# HCO_3_^−^/Cl^−^ Exchange Inactivation and Reactivation during Mouse Oocyte Meiosis Correlates with MEK/MAPK-Regulated Ae2 Plasma Membrane Localization

**DOI:** 10.1371/journal.pone.0007417

**Published:** 2009-10-12

**Authors:** Chenxi Zhou, Mario Tiberi, Binhui Liang, Seth L. Alper, Jay M. Baltz

**Affiliations:** 1 Ottawa Hospital Research Institute, Ottawa, Ontario, Canada; 2 Division of Reproductive Medicine, Department of Obstetrics and Gynecology, University of Ottawa Faculty of Medicine, Ottawa, Ontario, Canada; 3 Department of Cellular and Molecular Medicine, University of Ottawa Faculty of Medicine, Ottawa, Ontario, Canada; 4 Departments of Psychiatry and Medicine, University of Ottawa Faculty of Medicine, Ottawa, Ontario, Canada; 5 Molecular and Vascular Medicine Unit and Renal Division, Beth Israel Deaconess Medical Center, and Department of Medicine, Harvard Medical School, Boston, Massachusetts, United States of America; Sun Yat-Sen University, China

## Abstract

**Background:**

Germinal Vesicle (GV) stage mouse oocytes in first meiotic prophase exhibit highly active HCO_3_^−^/Cl^−^ exchange—a class of transport nearly ubiquitously involved in regulation of intracellular pH and cell volume. During meiosis, however, oocyte HCO_3_^−^/Cl^−^ exchange becomes inactivated during first metaphase (MI), remains inactive in second metaphase (MII), and is reactivated only after egg activation. Previous work using pharmacological manipulations had indicated that activity of the MEK/MAPK signaling pathway was negatively correlated with HCO_3_^−^/Cl^−^ exchange activity during meiosis. However, the mechanism by which the exchanger is inactivated during meiotic progression had not been determined, nor had the role of MEK/MAPK been directly established.

**Methodology/Principal Findings:**

Expression of a constitutively active form of MEK (MAP kinase kinase), which prevented the normal downregulation of MAPK after egg activation, also prevented reactivation of HCO_3_^−^/Cl^−^ exchange. Conversely, suppression of endogenous MAPK activity with dominant negative MEK activated the normally quiescent HCO_3_^−^/Cl^−^ exchange in mature MII eggs. A GFP-tagged form of the HCO_3_^−^/Cl^−^ exchanger isoform Ae2 (Slc4a2) was strongly expressed at the GV oocyte plasma membrane, but membrane localization decreased markedly during meiotic progression. A similar pattern for endogenous Ae2 was confirmed by immunocytochemistry. The loss of membrane-localized Ae2 appeared selective, since membrane localization of a GFP-tagged human dopamine D1 receptor did not change during meiotic maturation.

**Conclusions:**

Direct manipulation of MAPK activity indicated that GFP-tagged Ae2 localization depended upon MAPK activity. Inactivation of HCO_3_^−^/Cl^−^ exchange during the meiotic cell cycle may therefore reflect the loss of Ae2 from the oocyte plasma membrane, downstream of MEK/MAPK signaling. This identifies a novel role for MEK/MAPK-mediated cytostatic factor (CSF) activity during meiosis in membrane protein trafficking in mouse oocytes, and shows for the first time that selective retrieval of membrane proteins is a feature of meiosis in mammalian oocytes.

## Introduction

Maintenance of pH_i_ within a narrow physiological range is a fundamental property of almost every cell, since many key cellular processes are highly sensitive to pH [1]–[3]. In mammalian cells, pH_i_ is regulated against alkalosis mainly by Na^+^-independent, electroneutral HCO_3_^−^/Cl^−^ exchangers of the AE (anion exchanger) family [4]–[7], which act to correct pH_i_ increases by exporting HCO_3_^−^ in exchange for Cl^−^. AE HCO_3_^−^/Cl^−^ exchangers are members of the Solute Carrier 4 (*SLC4*) gene family, with Ae1 (Slc4a1), Ae2 (Slc4a2), Ae3 (Slc4a3) and possibly Ae4 (Slc4a9) established as HCO_3_^−^/Cl^−^ exchangers. In addition to pH_i_ regulation, AE family HCO_3_^−^/Cl^−^ exchangers also play key roles in cell volume regulation and epithelial fluid and ion transport [6], [8]. HCO_3_^−^/Cl^−^ exchange has been shown to be active in mammalian preimplantation embryos and oocytes, with activity in oocytes dependent on the meiotic cell cycle [9]–[12].

In mammals, fully-grown germinal vesicle (GV) stage oocytes are actively maintained in prophase arrest by high intracellular levels of cyclic adenosine monophosphate (cAMP) [13]. Release of prophase arrest and germinal vesicle breakdown (GVBD) are triggered by ovulation or by oocyte removal from the follicle, initiating progression through first meiotic metaphase (MI) into second meiotic metaphase (MII) to produce a mature egg that re-arrests in MII. Active maintenance of MII arrest is controlled by the oocyte-specific Cytostatic Factor (CSF), which requires activity of the mitogen-activated protein kinase (MAPK) pathway of the oocyte [14], [15]. Meiotic MAPK activity is controlled by the oocyte-specific protein kinase, MOS, which activates the MAPK kinase, MEK. MEK in turn activates ERK-type MAPK and downstream effectors that maintain M-phase promoting factor (MPF) activity [14]–[16]. MII arrest persists until fertilization (or parthenogenetic egg activation), when the egg completes meiosis and the MAPK pathway is inactivated, resulting in pronuclear formation and entry into interphase [17].

During its growth in the ovary, the oocyte is connected to surrounding ovarian granulosa cells (GC) through gap junctions, and fails to develop if these connections are disrupted [18]. Growing oocytes are dependent on GC for basic cellular functions, such as the provision of amino acids and metabolic substrates [19]–[22]. This dependence was recently shown to include intracellular pH (pH_i_) regulation, since isolated growing oocytes are unable to control their own pH_i_ [23] but instead rely on GC, which control oocyte pH_i_ through gap junction-mediated oocyte-GC communication [24], [25]. Only when the oocyte is nearly fully grown does it acquire the capacity to regulate its own pH_i_ [23].

Fully grown GV stage mouse oocytes possess robust HCO_3_^−^/Cl^−^ exchanger activity that regulates pH_i_ and confers protection against alkalosis [11], [23]. HCO_3_^−^/Cl^−^ exchange is also highly active in preimplantation (PI) mammalian embryos from the mid-1-cell through the blastocyst stages [10], [12], [26]–[28], and is required for mouse embryos to maintain pH_i_ and for normal embryo development [10], [29]. Of the three AE HCO_3_^−^/Cl^−^ exchangers, Ae1–3 [30], both Ae2 and Ae3 mRNAs are expressed in mouse PI embryos starting at the 2-cell stage, while only Ae2 is present in eggs and 1-cell embryos [28], [29], [31]. Thus, pH_i_-regulatory HCO_3_^−^/Cl^−^ exchanger is active in GV oocytes and 1-cell embryos and is most likely mediated by Ae2.

Despite high HCO_3_^−^/Cl^−^ exchange activity before meiotic maturation and in 1-cell embryos after fertilization, MII eggs surprisingly exhibit almost no HCO_3_^−^/Cl^−^ exchanger activity [10], [11], [28]. HCO_3_^−^/Cl^−^ exchange was shown to inactivate gradually during MI and to remain nearly quiescent in MII [11], [28], [32]. Egg activation triggers reactivation of anion exchange only after several hours, with maximal activity achieved around the time of pronuclei formation.

The mechanism by which HCO_3_^−^/Cl^−^ exchanger activity is inactivated and reactivated during the meiotic cell cycle has not been established. However, we previously demonstrated that the timecourse of HCO_3_^−^/Cl^−^ exchanger inactivation and reactivation closely followed MAPK activity. Pharmacological manipulations indicated a negative correlation between MAPK and HCO_3_^−^/Cl^−^ exchanger activities during meiosis, in contrast to the lack of correlation with MPF activity [11], [15]. These results suggested that the MAPK pathway might regulate HCO_3_^−^/Cl^−^ exchanger activity during meiosis [11], [33]. However, this had not been directly established nor was the mechanism of regulation known.

In this study, we have directly manipulated MEK and MAPK activities during meiosis by expressing constitutively active and dominant negative forms of MEK, to determine whether HCO_3_^−^/Cl^−^ exchanger activity is indeed negatively regulated by MAPK. We also have tested the hypothesis that inactivation of HCO_3_^−^/Cl^−^ exchange during meiosis is due to retrieval of Ae2 from the plasma membrane. Our results indicate a novel regulation of HCO_3_^−^/Cl^−^ exchanger activity and Ae2 trafficking by MEK/MAPK in the meiotic cell cycle.

## Materials and Methods

### Ethics statement

Animal protocols were approved by the Animal Care Committee of the Ottawa Hospital Research Institute.

### Chemical and solutions

All chemicals were obtained from Sigma (St Louis, MO) unless otherwise noted. Stock solutions (1000×) were prepared in water for dibutyryladenosine 3,5-cyclic monophosphate (dbcAMP) and cycloheximide, in ethanol for nigericin, and in dimethyl sulfoxide (DMSO) for valinomycin and carboxyseminaphthorhodafluor-1-acetoxymethyl ester (SNARF-1-AM; Invitrogen Molecular Probes, Eugene, OR), and were stored at −20°C.

Culture media used during experimental manipulations were based on KSOM mouse embryo medium (Lawitts and Biggers, 1993), which contains (in mM) 95 NaCl, 2.5 KCl, 0.35 KH_2_PO_4_, 0.2 MgSO_4_, 10 Na lactate, 0.2 glucose, 0.2 Na pyruvate, 25 NaHCO_3_, 1.7 CaCl_2_, 1 glutamine, 0.01 tetra sodium EDTA, 0.03 streptomycin SO_4_ and 0.16 penicillin G, and 1 mg/ml bovine serum albumin (BSA). HCO_3_^−^/CO_2_-buffered media were equilibrated with 5% CO_2_/air. HEPES-KSOM (21 mM HEPES replacing equimolar NaHCO_3_, pH adjusted to 7.4) was used for oocyte collection and microinjection. For pH_i_ measurements, all but 1 mM Na lactate was replaced with NaCl, and BSA was omitted. Cl^−^-free media were produced by replacing Cl^−^ salts with corresponding gluconate salts. HCO_3_^−^-free media were used in which 21 mM of NaHCO_3_ was replaced by HEPES and the remainder by NaCl, with pH adjusted to 7.4. For expression of exogenous proteins in oocytes, cRNA-microinjected GV oocytes (below) were incubated in MEMα medium (12561*-*056; Invitrogen, San Diego, CA) supplemented with 0.16 mM penicillin G, 0.03 mM streptomycin sulfate, and 1 mg/ml PVA. Where specified, GV oocytes were maintained in GV arrest by supplementing the medium with 300 µM dbcAMP, which completely and reversibly maintains arrest of mouse GV oocytes [11].

### Oocyte collection, maturation, and parthenogenetic activation

GV oocytes were obtained from primed CF1 female mice (6–8 weeks; Charles River, St-Constant, PQ, Canada), approximately 44 hours after equine chorionic gonadotropin (eCG) injection (5 IU, intraperitoneally). Ovaries were removed and minced to release cumulus-enclosed oocytes in HEPES-KSOM with dbcAMP. Cumulus cells were subsequently removed by repeated pipetting through a narrow-bore pipette. In vivo-derived MII eggs were obtained from females injected with human chorionic gonadotropin (hCG, 5 IU, intraperitoneally) ∼47 hours post-eCG. MII eggs were collected from excised oviducts at ∼17 hours post-hCG (eggs retrieved at this time, which is several hours after fertilization would take place in vivo, respond optimally to parthenogenetic activation). One-cell zygotes were obtained from females treated as for MII eggs that were then caged individually overnight with BDF1 males. Zygotes were collected from excised oviducts at ∼22 hours post-hCG.

When cRNA microinjection was carried out (below), GV oocytes were washed after microinjection 3 times in warm MEMα with dbcAMP and then cultured in the same medium at 37°C, in an atmosphere of 5% CO_2_ in air, for 20 hours to allow expression. GV oocytes expressing exogenous proteins were then used for experiments either maintained in GV arrest by the continued presence of dbcAMP, or during spontaneous meiotic progression induced by the removal of dbcAMP. To obtain MII eggs expressing exogenous proteins, oocytes were transferred to dbcAMP-free MEMα medium at 5 hours after microinjection and then cultured for another 15 hours so that the total time for expression was identical to that for GV oocytes (20 hours). Parthenogenetic activation of mouse MII oocytes, including those expressing exogenous proteins, was performed as previously reported [11]. Briefly, MII eggs were incubated for 2 hours in KSOM with CaCl_2_ omitted and 10 mM SrCl_2_ added [34], [35]. After activation, eggs were washed and transferred to normal KSOM.

### cRNA preparation and microinjection

Constitutively active mouse MEK1 (MEK-EE; serines 218 and 222 mutated to glutamate, NCBI Reference Sequence: NM_008927.3) in pCDNAIIIB vector [36], [37] was a gift of Dr. Silvio Gutkind (NIH, USA). Dominant negative human MEK1 (MEK-AA; serines 218 and 222 mutated to alanine, NCBI Reference Sequence: NM_002755) [38], [39] was a gift of Dr. Rony Seger (The Weizmann Institute of Science, Israel), and was subcloned into pCDNAIII vector (Invitrogen). Human MEK1 has 98% amino acid sequence identity to mouse MEK1. Ae2-GFP (in pXT7 vector, NCBI Reference Sequence: NM_009207.2) was previously described and shown to be functional [40]. GFP plasmid [41] was a gift from Dr. Johné Liu (Ottawa Health Research Institute, Canada). A full-length version of the human dopamine D1 receptor (hD1) with the cytoplasmic tail fused to GFP (hD1-GFP) was subcloned into cDNA3.1 (Invitrogen) and the sequence confirmed by direct sequencing. Plasma membrane localization and functionality of hD1-GFP was validated in transfected human embryonic kidney 293 (HEK293) cells using radioligand binding, whole cell cAMP assays and indirect immunofluorescence microscopy (data not shown) as previously described [42], [43]. In all cases, cRNA was prepared from linearized cDNA templates (mMESSAGE mMACHINE; Ambion, Austin, TX) resuspended in RNAse-free water and stored at −80°C until microinjected. cRNA size and integrity were confirmed by gel electrophoresis.

cRNA microinjection was performed in drops of HEPES-KSOM under oil using a Medical Systems PLI-100 microinjection apparatus (Harvard Apparatus, Holliston, MA) controlling holding and injection pipets, with Narishige MMN-1 micromanipulators mounted on a Zeiss Axiovert microscope. Each oocyte was microinjected with 10 pl of 1 µg/µl in vitro transcribed cRNA. For each cohort, 15–20 oocytes were injected within 30 minutes of oocyte collection.

### pH_i_ measurements and Cl^−^ removal assay for HCO_3_^−^/Cl^−^ exchange activity

pH_i_ measurements were performed using a quantitative imaging microscopy system (ISee Imaging Systems, Raleigh, NC) as previously described [24]. The pH-sensitive fluorophore, SNARF-1 was loaded intracellularly by incubation with its acetoxymethyl ester derivative (SNARF-1-AM, 5 µM, 30 minutes, 37°C). pH_i_ was determined in SNARF-1-loaded oocytes or eggs using fluorescence ratio imaging of images at emission wavelengths of 640 and 600 nm with an excitation wavelength of 535 nm. The ratio of the two intensities (640/600), which is dependent mainly on pH_i_, was calculated by dividing the fluorescence intensities measured individually over each egg after background subtraction. Calibration was performed using the nigericin/high K^+^ method [28]. All measurements were performed in a temperature- and atmosphere-controlled chamber (37°C, 5% CO_2_/air).

HCO_3_^−^/Cl^−^ exchanger activity was quantified by the Cl^−^ removal method [12], [27], [28]. Upon exposure of cells to Cl^−^ free solution, any active HCO_3_^−^/Cl^−^ exchanger will run in reverse, resulting in intracellular alkalinization due to HCO_3_^−^ influx coupled to Cl^−^ efflux [44]. Increased pH_i_ upon Cl^−^ removal thus indicates HCO_3_^−^/Cl^−^ exchanger activity, with the initial rate of alkalinization providing a quantitative measure. Here, SNARF-1-loaded eggs or oocytes were placed in the chamber, allowed to equilibrate for 15 minutes, and then pH_i_ measurements obtained for 10 minutes, after which the solution was changed to Cl^−^ free medium and measurements continued for 20 minutes. The initial rate of intracellular alkalinization upon Cl^−^ removal was determined in each individual oocyte or egg using linear regression (SigmaPlot 8.0; SPSS, Chicago, IL), and reported as the change in pH_i_ per minute (pH U/min). Inhibition by DIDS (500 µM) confirmed that any observed alkalinization was mediated by HCO_3_^−^/Cl^−^ exchange. Simultaneous measurements were made on groups of 5–25 oocytes. This assay for HCO_3_^−^/Cl^−^ exchanger activity has been extensively described and validated in mouse oocytes and embryos [11], [23]–[25], [28].

### Measurement of GFP-tagged protein expression and membrane localization

Images of GFP-expressing oocytes were obtained with a TMD fluorescence microscope (Nikon, Tokyo, Japan) with 488 nm excitation and 510 nm emission, using a QImaging CCD camera with Qcapture image software (Qimage Corp. Surrey, BC, Canada). ImageJ 1.4 software (NIH, Bethesda, MD) was used for quantitative analysis of plasma membrane vs. cytoplasmic localization. Background taken just outside the cells was subtracted from each image. Rectangular regions of interest were placed at four points (separated by 90°) around the oocyte circumference, with each region of interest spanning the plasma membrane from outside the cell into the cytoplasm. Average intensity within each region of interest was determined as a function of radial distance in each of the four regions per oocyte. To calculate ratios of fluorescence intensity at the plasma membrane to that in the adjacent cytoplasm, the intensity at the plasma membrane was divided by the average intensity starting 5 pixels inside the oocyte and extending a distance of 10 pixels into the oocyte.

### Measurement of MAPK activity

The MBP kinase activity assay was modified from Moos et al. (1996) as previously described and validated [11]. Briefly, seven washed eggs along with ∼1–1.5 µl medium were added to 3.5 µl lysis buffer [11] and stored at −80°C. The kinase assay reaction was initiated by thawing and addition of 5 µl kinase buffer [11] containing 500 µCi/ml ^32^P-ATP (Amersham, Pharmacia Biotech, NJ, USA). After 30 minutes, the reaction was stopped by boiling after addition of 10 µl 2× Laemmli sample buffer, and the reaction products then separated by 15% SDS-PAGE. ^32^P-phosphorylated MBP was quantified with a Phosphorimager (Typhoon 8600 with ImageQuant software; Molecular Dynamics, Inc., Sunnyvale, CA). Background, determined from a lane containing a reaction run without eggs, was subtracted. A reaction run with fresh MII eggs was included in each gel, and MBP kinase activities were expressed relative to the values for eggs (set to 100%) within each gel. MBP phosphorylation was assumed to indicate MAPK activity as previously demonstrated [11], [36].

### Ae2 immunocytochemistry

Oocytes or eggs were fixed in 2% formaldehyde in PBS for 40 minutes at 37°C after the zona pellucidae (ZP) had been removed by brief exposure to acid Tyrode's (pH 2.5). Ae2 was detected by whole-mount immunocytochemistry using an affinity-purified rabbit polyclonal antibody. The antiserum raised against a peptide corresponding to amino acids 1224–1237 in the mouse Ae2 C-terminus was affinity-purified with the immunogenic peptide [45], and resuspended to 0.5 mg/ml IgG in 1% BSA with 0.02% Na azide. Since antigen detection by this antibody requires epitope unmasking [46] in some tissues, fixed oocytes or eggs were treated with 1% SDS for 2.5 minutes and washed. Oocytes or eggs were then incubated in blocking solution (2% BSA, 2% FBS in PBS; 1 hour, 37°C) followed by overnight incubation at 4°C with the Ae2 antibody (1∶500). Oocytes or eggs were then incubated with goat anti-rabbit Alexa 488 secondary antibody (Invitrogen Molecular Probes; 3 hours, 37°C) before mounting. Confocal images were collected using an Olympus IX70 inverted microscope equipped with a BioRad MRC-1024 confocal laser-scanning unit, as described previously (Ma et al., 2006), using 488 nm excitation and 520 nm emission. Typically, 30–40 images were collected at 2 µm intervals through each oocyte or egg. Each experiment was repeated three times, and at least 30 eggs were examined in each group with the same instrument settings used for each repeat.

### Data analysis

Data are presented as the mean±the standard error of the mean (s.e.m.). N indicates the number of separate experiments, while n indicates the total number of oocytes or eggs. Means of the data were compared using *t*-tests (two groups) or ANOVA (three or more groups). Where ANOVA was used, Tukey-Kramer's post-hoc test was applied (Prism 3, GraphPad Software Inc., San Diego, CA). In all cases, P<0.05 was considered significant.

## Results

### Regulation of HCO_3_^−^/Cl^−^ exchanger activity downstream of MEK/MAPK during meiotic maturation of mouse oocytes

**Figure 1:**
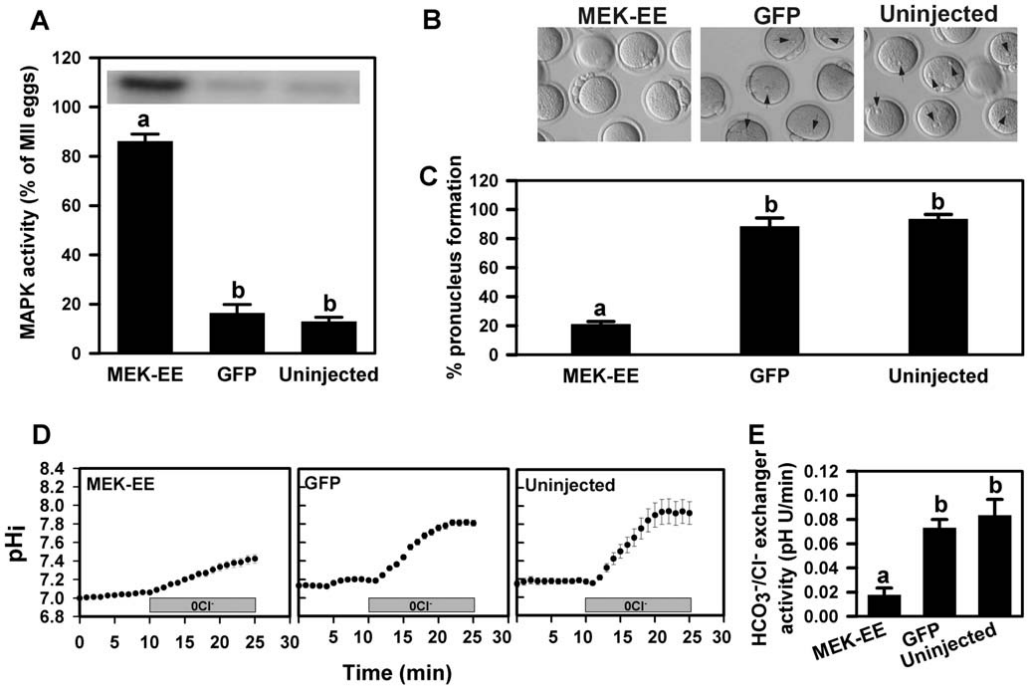
Effect of constitutively active MEK expression on MAPK and HCO_3_^−^/Cl^−^ exchanger activities in activated eggs. (A) Mean (±s.e.m.) MAPK activities in Sr^2+^-activated eggs expressing MEK-EE or GFP, or in uninjected controls at 5 hours post-Sr^2+^ (N = 3). The inset shows a representative example of a MBP kinase assay gel. Activity (extent of phosphorylation as determined from densities of bands on gel) is expressed relative to that of uninjected MII oocytes that were included in each assay (a lane with unfertilized eggs was run on each gel to determine maximal MAPK activity, with the density of that band considered 100%; the unfertilized egg band is not shown here). (B) Representative images of activated eggs from each treatment group (indicated by labels at top). Arrows indicate the presence of pronuclei in GFP-injected and uninjected groups whereas no pronuclei formed in MEK-EE group. (C) Percent pronuclear formation in eggs expressing MEK-EE or GFP, or uninjected controls at 5 hours post-Sr^2+^ (N = 3–4; 20–30 eggs in each group). (D) Representative examples of measurements of HCO_3_^−^/Cl^−^ exchanger activity at 5 hours post-Sr^2+^ using the Cl^−^ removal assay (presence of Cl^−^-free solution indicated by gray boxes). Traces show mean (±s.e.m.) pH_i_ as a function of time in a group of 20–30 uninjected eggs measured simultaneously. (E) Mean (±s.e.m.) HCO_3_^−^/Cl^−^ exchanger activities at 5 hours post- Sr^2+^ from measurements like those shown in (D), with activity measured as initial slope upon Cl^−^ removal (N = 3–4; 20–30 eggs in each group). Bars in A,C and E that do not share the same letter indicate within a panel significant difference as evaluated by one-way ANOVA and Tukey-Kramer Multiple Comparisons Test (*P*<0.001 in A and C; *P*<0.05 in E).

Previous pharmacological data indicated that MAPK might negatively regulate HCO_3_^−^/Cl^−^ exchanger activity during meiosis. Thus, one prediction would be that expression of constitutively activated MEK in parthenogenetically-activated eggs would prevent the normal reactivation of HCO_3_^−^/Cl^−^ exchange. We expressed MEK-EE, a constitutively activated form of MEK [36], [37], in mouse eggs to prevent the inactivation of MAPK that normally occurs several hours after egg activation. Uninjected oocytes and oocytes injected with cRNA encoding GFP served as controls. Oocytes were removed from dbcAMP, in vitro matured to the MII egg stage, and parthenogenetically activated with Sr^2+^. The activation of the eggs from each group was confirmed by the emission of a second polar body (indicating inactivation of MPF). Maintenance of high MAPK activity in MEK-EE-injected eggs, but not uninjected or GFP-injected control eggs, after parthenogenetic activation was confirmed by MBP kinase assay at 5 hours post-activation (Figure 1A). Since it was previously reported that high MAPK activity is incompatible with the formation of pronuclei [17], [36], we also assessed pronuclear formation and confirmed that formation of pronuclei was inhibited in MEK-EE -injected eggs compared to GFP-injected or uninjected eggs at 5 hours post-activation (Figure 1B,C). Therefore, MAPK activity was maintained in activated eggs by MEK-EE.

We then determined whether MEK-EE expression affected HCO_3_^−^/Cl^−^ exchanger activity in parthenogenotes using pH_i_ measurements on SNARF-1-loaded oocytes with the Cl^−^ removal assay. HCO_3_^−^/Cl^−^ exchange is normally reactivated within 5 hours after egg activation [11]. We found that MEK-EE substantially reduced the appearance of HCO_3_^−^/Cl^−^ exchanger activity at 5 hours post-activation, while exchanger activity had developed normally in GFP-injected or uninjected eggs (Figure 1D, E). Thus, HCO_3_^−^/Cl^−^ exchange reactivation after egg activation was prevented by MEK-EE expression that maintained high MAPK activity.

**Figure 2:**
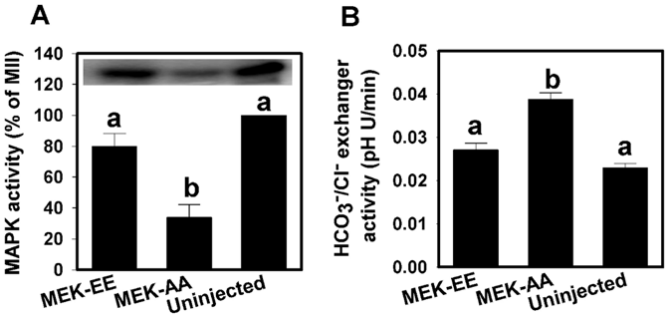
Effect of dominant negative MEK expression on MAPK and HCO_3_^−^/Cl^−^ exchanger activities in oocytes during meiotic maturation. (A) Mean (±s.e.m.) MAPK activity in MII eggs expressing MEK-EE, MEK-AA, or in uninjected controls (N = 3). The inset shows a representative example of a MBP kinase assay gel. Activity is expressed as percent of activity in uninjected MII oocytes on same gel (not shown). (B) Mean (±s.e.m.) HCO_3_^−^/Cl^−^ exchanger activities in MII eggs expressing MEK-EE, MEK-AA, or in uninjected controls (N = 3; 20–30 eggs per group). Bars that do not share the same letter within each panel indicate significant difference as evaluated by one-way ANOVA and Tukey-Kramer Multiple Comparisons Test (*P*<0.01).

A second prediction would be that suppressing MEK activity in oocytes during meiotic progression would prevent the inactivation of HCO_3_^−^/Cl^−^ exchange during MI and result in MII eggs with abnormally high exchanger activity. To test this, we expressed MEK-AA, a dominant negative form of MEK [38], [39], during meiotic maturation. Uninjected oocytes and oocytes injected with cRNA for MEK-EE (which should have no effect on MAPK in MII eggs since it is already maximally activated) served as controls. After cRNA microinjection at the GV stage, injected and control oocytes were in vitro matured overnight to the MII stage. Substantial suppression of MAPK activity in MEK-AA-expressing MII eggs but not uninjected or MEK-EE-injected controls was confirmed by MBP kinase assay (Figure 2A). Measurement of HCO_3_^−^/Cl^−^ exchanger activity by the Cl^−^ removal assay indicated similar low exchanger activity in uninjected MII eggs and eggs expressing MEK-EE. MEK-AA-expressing MII eggs, however, exhibited significantly higher HCO_3_^−^/Cl^−^ exchanger activity (Figure 2B).

### Ae2 membrane localization during meiotic maturation

**Figure 3:**
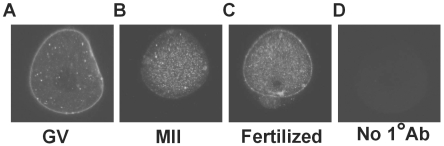
Immunofluorescence localization of Ae2 in mouse oocytes during meiotic maturation. (A) Representative confocal images through the center of a GV oocyte (A), MII egg (B), and fertilized egg at 22 hours post-hCG (C) showing Ae2 immunofluorescence. (D) GV oocyte treated identically to that in (A) except the primary antibody was omitted. Each oocyte or egg shown is representative of 20–30 total at each stage in three independent repeats.

We used an antibody specific for the Ae2 HCO_3_^−^/Cl^−^ exchanger expressed in oocytes to examine its subcellular localization in in vivo-derived GV oocytes, MII eggs, and fertilized eggs (22 hours post-hCG). Confocal microscopy showed that Ae2 immunofluorescence in GV oocytes was localized predominantly to the plasma membrane (Figure 3A), consistent with high HCO_3_^−^/Cl^−^ exchanger activity observed at that stage. However, in MII eggs, comparatively little Ae2 immunostaining was localized at the membrane (Figure 3B). In fertilized eggs, increased membrane-localized Ae2 immunostaining was again evident (Figure 3C). These results show that subcellular localization of Ae2 in mouse oocytes varies during meiotic progression, and that membrane localization of Ae2 is low in MII eggs relative to GV oocytes or fertilized eggs.

**Figure 4:**
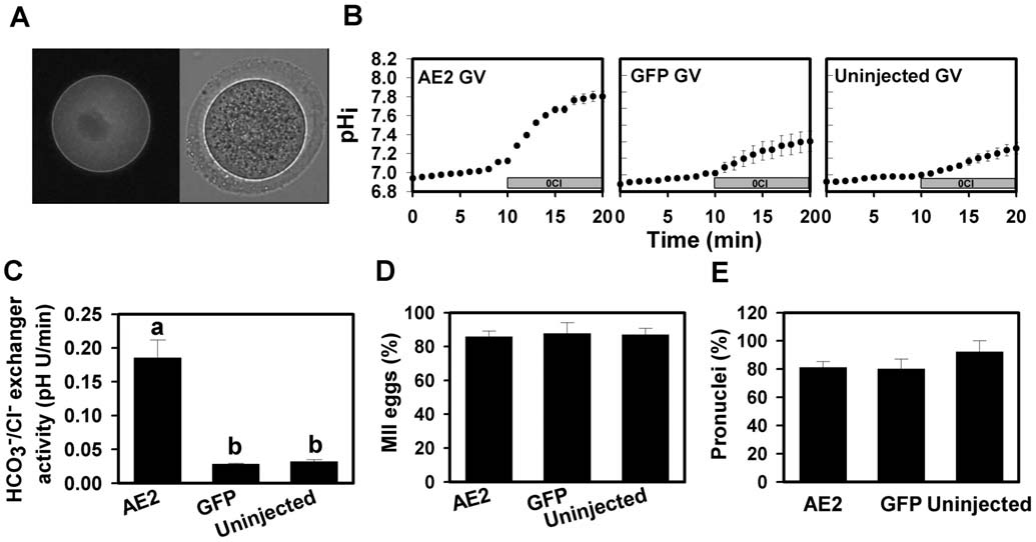
Expression and function of Ae2-GFP in live oocytes. (A) Representative fluorescence image of a GV-arrested oocyte (in the presence of dbcAMP) expressing Ae2-GFP at 20 hours post-cRNA injection (left) and phase-contrast image (right). (B) Representative examples of measurements of HCO_3_^−^/Cl^−^ exchanger activity by the Cl^−^ removal assay (as in Fig. 1) for each treatment group (indicated by labels in panels). (C) Mean (±s.e.m.) HCO_3_^−^/Cl^−^ exchanger activity in GV-arrested oocytes expressing Ae2-GFP (20 hours post-injection), GFP or uninjected oocytes. Different letters indicate significant difference (N = 3–4 repeats; P<0.001; ANOVA and Tukey-Kramer Multiple Comparisons Test). D) Mean (±s.e.m.) percentage of GV oocytes expressing AE2-GFP, GFP, or uninjected controls progressing to MII after removal from dbcAMP and continued culture for another 15 hours (N = 3; not significantly different by ANOVA). (E) Mean (±s.e.m.) percentage of parthenogenotes expressing AE2-GFP, GFP, or uninjected controls forming pronuclei at 5 hours post-Sr^2+^ activation (N = 3; not significantly different by ANOVA).

To examine further the dynamics of Ae2 distribution during oocyte maturation, we expressed a functional GFP-mouse Ae2 fusion protein (Ae2-GFP) in mouse oocytes. We confirmed that Ae2-GFP was present in the plasma membrane of GV oocytes that were kept arrested for 20 hours (with dbcAMP) to permit expression (Figure 4A). We also confirmed that Ae2-GFP was functional, using the Cl^−^-removal assay to show that HCO_3_^−^/Cl^−^ exchanger activity was greatly increased in GV oocytes expressing Ae2-GFP, but not in control GV-arrested oocytes that were uninjected or injected with cRNA for GFP alone (Figure 4B,C). Furthermore, expression of Ae2-GFP did not disrupt meiotic maturation to the MII stage when arrest was released (Figure 4D), or the development of pronuclei after parthenogenetic egg activation (Figure 4E). We noted that the HCO_3_^−^/Cl^−^ exchanger activity in the control GV-arrested oocytes was lower than those of fresh uninjected GVs (usually ∼0.05–0.08 pHU/min), possibly due to a decrease in endogenous HCO_3_^−^/Cl^−^ exchanger activity with extended GV arrest (∼20 hr) and maintenance in culture.

**Figure 5:**
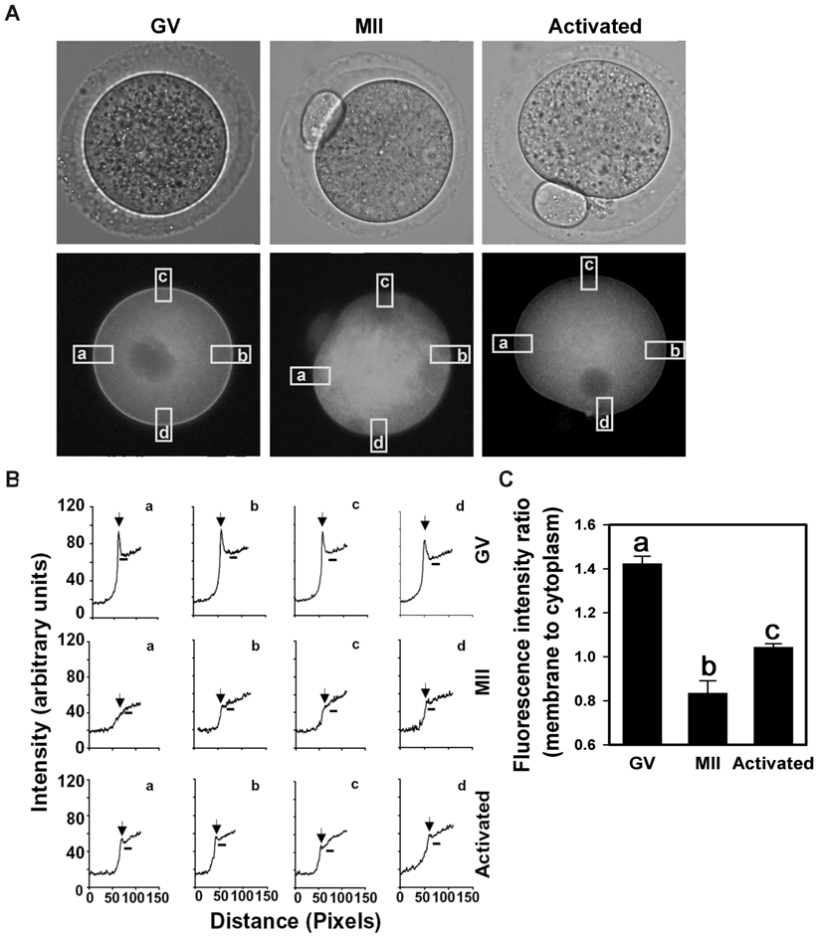
Ae2-GFP localization in mouse oocytes during meiotic maturation. (A) Representative fluorescence (lower panel) and phase-contrast images (upper panel) of GV oocytes, MII eggs and Sr^2+^-activated eggs expressing Ae2-GFP. The rectangles in the fluorescence images are the regions of interest within which intensities were measured. (B) Fluorescence intensities as a function of radial distance starting outside the oocyte measured within the regions of interest indicated by the rectangles on images in A. The labels a, b, c, and d indicate measurements for the regions of interest with the same label in A. The oocyte for which each set of four measurements is shown is indicated at right. Arrows indicate position of plasma membrane. The transition from outside to inside of the oocyte is plotted from left to right as a function of radial distance in pixels. Cytoplasmic fluorescence intensities were calculated as the average within the range (10 pixels) indicated by the lines on each graph. (C) Mean (±s.e.m.) plasma membrane-to-cytoplasm fluorescence ratios for GV oocytes, MII eggs and activated eggs (N = 3 repeats with a total of 8–11 at each stage). Different letters above bars indicate significant difference (ANOVA and Tukey-Kramer Multiple Comparisons Test; *P*<0.001).

We then compared the localization of Ae2-GFP in GV oocytes, MII eggs, and parthenogenetically-activated eggs. GV oocytes and MII eggs were examined after the same length of overnight culture with or without dbcAMP, respectively, while parthenogenetically-activated eggs were a cohort of the MII eggs that were then Sr^2+^-activated and examined 5 hours later. A strong fluorescent signal was observed near the plasma membrane of Ae2-GFP-expressing GV oocytes (Figure 5A left). In MII eggs, Ae2-GFP was predominantly localized intracellularly, with an apparently lower GFP signal in the membrane as compared to GV oocytes (Figure 5A middle). The relative lack of membrane localization of Ae2-GFP could not be attributed to low expression levels, because increasing the amount of injected Ae2-GFP cRNA by 2–3 fold consistently produced the same distribution pattern in MII eggs, despite much higher overall fluorescence (not shown). At 5 hours after parthenogenetic activation, Ae2-GFP fluorescence was again apparent localized to the plasma membrane (Figure 5A right).

**Figure 6:**
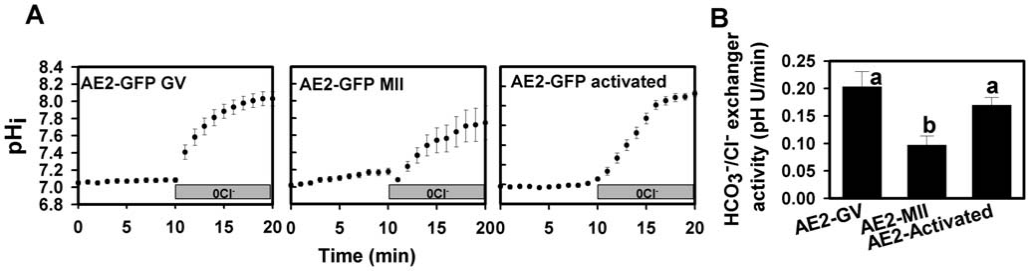
HCO_3_^−^/Cl^−^ exchanger activity in Ae2-GFP expressing oocytes during meiotic maturation. (A) Representative examples of measurements of HCO_3_^−^/Cl^−^ exchanger activity by the Cl^−^ removal assay in Ae2-GFP expressing GV oocytes, MII eggs and activated eggs. (B) Mean (±s.e.m.) HCO_3_^−^/Cl^−^ exchanger activities of treatment groups as in A (N = 3). Different letters indicate significant difference (ANOVA and Tukey-Kramer Multiple Comparisons Test; *P*<0.05).

To obtain a quantitative measure of the subcellular distribution pattern of Ae2-GFP, fluorescence intensities across four orthogonal regions of interest from each oocyte were measured (example in Figure 5A,B), and the ratio of peak fluorescence (arrows in Figure 5B) to average fluorescence in an area just inside the plasma membrane (short lines in Figure 5B) was calculated. This showed that the mean membrane-to-cytoplasmic intensity ratios measured in GV oocytes and activated eggs were significantly higher than those in MII eggs (Figure 5C). The observed changes in Ae2-GFP membrane localization were reflected in measured HCO_3_^−^/Cl^−^ exchanger activity, with very high activity in Ae2-GFP-expressing GV oocytes and activated eggs, but significantly lower activity in MII eggs (Figure 6A,B).

**Figure 7:**
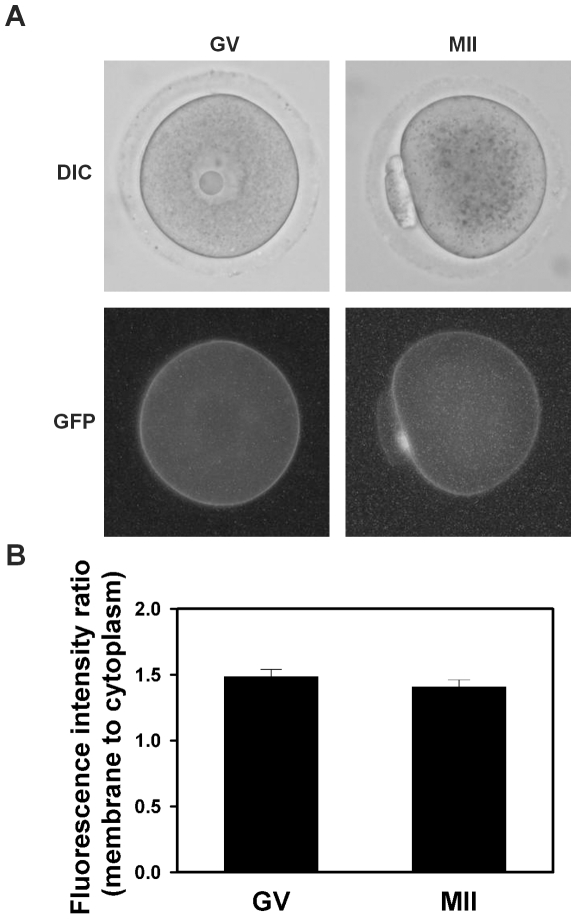
Localization of hD1-GFP in mouse oocytes during meiotic maturation. (A) Representative example of hD1-GFP-expressing GV oocyte and MII egg as indicated at top showing fluorescence (lower panel) and corresponding phase-contrast image (upper panel). (B) Mean (±s.e.m.) plasma membrane-to-cytoplasm fluorescence ratios of hD1-GFP-expressing GV oocytes and MII eggs (N = 4, with 17 images analyzed in each group; not significantly different by student's two-tailed t test).

To determine if the loss of membrane localization following resumption of meiosis was specific to Ae2, rather than a more general phenomenon affecting all membrane proteins (e.g., general endocytosis of plasma membrane or degradation of transmembrane proteins), we expressed in mouse oocytes an exogenous control membrane protein, human dopamine D1 receptor tagged with GFP (hD1-GFP), whose localization is not normally regulated by MAPK. The ratio of membrane-to-cytoplasmic fluorescence intensity in hD1-GFP-expressing GV and in MII eggs did not differ (Figure 7), suggesting, in contrast to Ae2-GFP, that the membrane localization of hD1-GFP did not change throughout oocyte maturation.

### Effect of MEK/MAPK activity on Ae2 membrane localization

**Figure 8:**
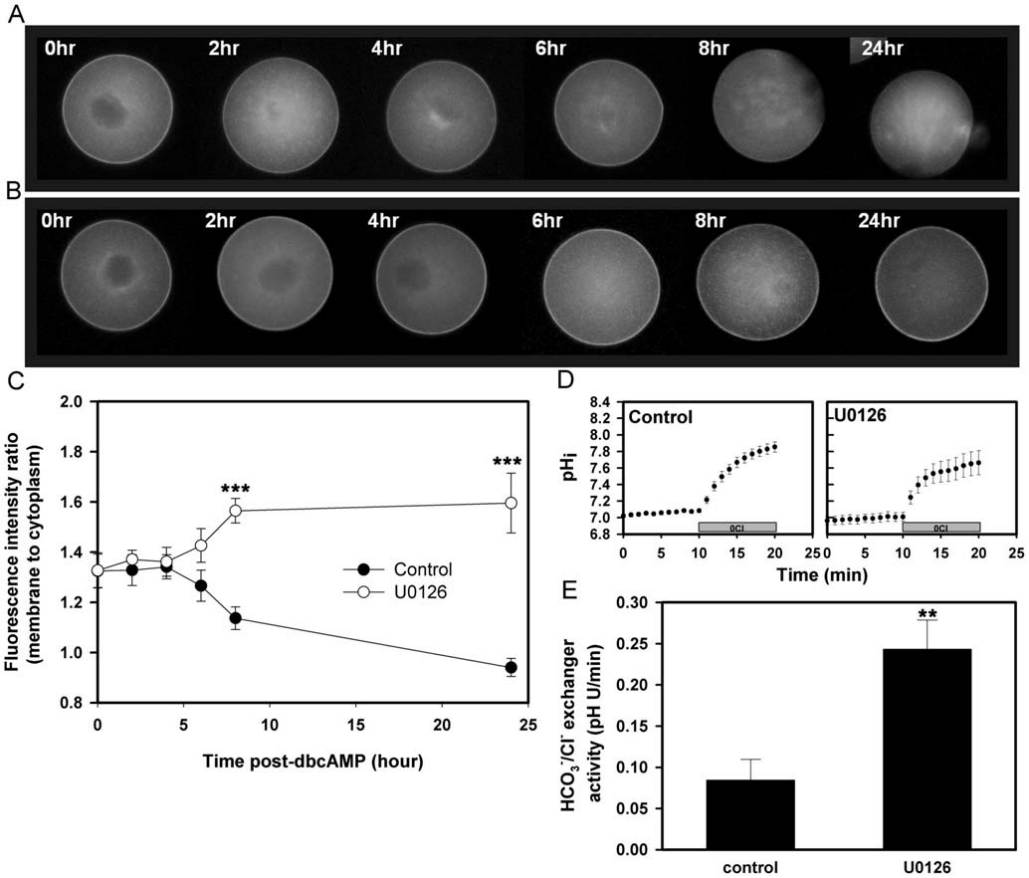
Effect of the MEK inhibitor, U0126, on Ae2-GFP localization and HCO_3_^−^/Cl^−^ exchanger activity in Ae2-GFP-expressing oocytes during meiotic maturation. Ae2-GFP expressing oocytes were induced to undergo spontaneous GVBD and meiotic maturation by removal from dbcAMP (t = 0) and fluorescence images obtained at the times indicated (hr = hours). Representative fluorescence images are shown of oocytes that were treated with 0.01% DMSO vehicle alone (A) or U0126 (20 µM) (B). (C) Mean (±s.e.m.) plasma membrane-to-cytoplasm Ae2-GFP fluorescence ratios of control and U0126-treated oocytes (as indicated by inset key) as a function of time after dbcAMP removal are shown (N = 3, totally 5–13 oocytes images were quantified for each time point). ***P<0.001 (Student's two-tailed t test for the two measurements at each time). (D) Representative examples of measurements of HCO_3_^−^/Cl^−^ exchanger activity by the Cl^−^ removal assay in Ae2-GFP-expressing oocytes treated with DMSO (control) or U0126 for 8 hours starting at dbcAMP removal. (E) Mean (±s.e.m.) HCO_3_^−^/Cl^−^ exchanger activities of treatment groups as in D (N = 3; 20–30 eggs per group; **P<0.01; Student's two-tailed t test).

The above results led to the hypothesis that MEK/MAPK activity regulates subcellular localization of Ae2, and mediates the observed changes in HCO_3_^−^/Cl^−^ exchanger activity during meiosis. Thus, inhibition of MEK/MAPK activity during meiotic maturation should maintain Ae2-GFP plasma membrane localization. We tested this hypothesis with U0126, a specific MEK inhibitor previously used to investigate the potential role of MEK/MAPK in endogenous HCO_3_^−^/Cl^−^ exchanger activity in oocytes during meiosis [15]. The use of U0126 allows the rapid and complete inactivation of MEK (in contrast to MEK-AA, which requires an extensive period for expression and did not completely inactivate MAPK). Ae2-GFP expressing GV oocytes were released from GV arrest (by removal of dbcAMP) in the presence of U0126 (20 µM; U0126 has been shown to be specific and reversible at concentrations of up to 50 µM in oocytes [15]), and fluorescence images obtained of oocytes in cohorts taken from the pool at time points from 0–8 hours and at 24 hours after removal from dbcAMP. Before 6 hours post-dbcAMP removal, there was no significant difference in the membrane-to-cytoplasm fluorescence ratio between U0126-treated oocytes and controls (0.1% DMSO). However, by 8 hours, the ratio in control oocytes had become significantly lower than in the presence of U0126, a difference that persisted at 24 hours (Figure 8A,B,C). We also confirmed that HCO_3_^−^/Cl^−^ exchanger activity in oocytes at 8 hours post-dbcAMP removal was significantly higher in the presence of U0126 than in control oocytes (Figure 8D,E).

**Figure 9:**
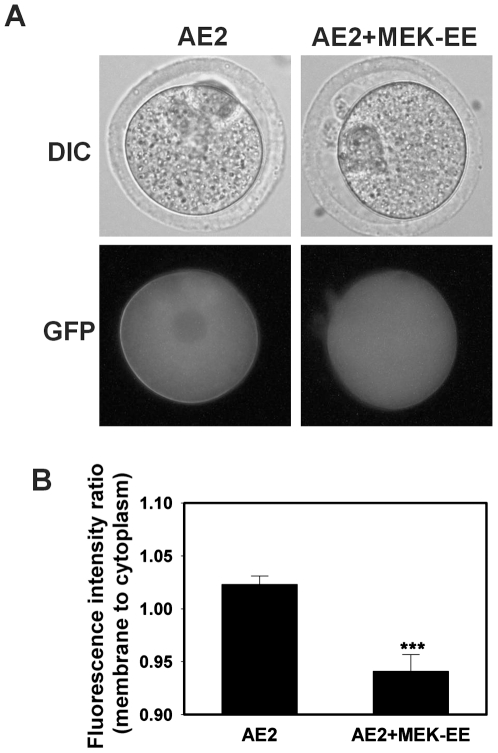
Effect of MEK-EE on membrane localization of Ae2-GFP in eggs following Sr^2+^ activation. A) Representative examples of fluorescence (lower panel) and corresponding phase-contrast images (upper panel) of Sr^2+^-activated eggs expressing Ae2-GFP only (left) or both MEK-EE and Ae2-GFP mRNA (right), at 5 hours after Sr^2+^ activation. (B) Mean (±s.e.m.) plasma membrane-to-cytoplasm fluorescence ratios of activated eggs as in A (N = 4, 36–50 oocyte images were quantified in each group; *** P<0.001; Student's two-tailed t test).

Another prediction is that maintaining MEK/MAPK activity after egg activation would prevent reappearance of Ae2-GFP at the membrane. We parthenogenetically activated (with Sr^2+^) eggs that had been in vitro-matured from GV oocytes co-injected with Ae2-GFP and MEK-EE. Fluorescence images of the activated eggs from each group were acquired at 5 hours post-activation, a time sufficient for MAPK inactivation. Expression of MEK-EE resulted in plasma membrane-to-cytoplasm fluorescence ratios significantly lower in eggs co-expressing MEK-EE/Ae2-GFP than in control eggs expressing only Ae2-GFP (Figure 9). The results suggested that elevated MAPK activity prevented Ae2-GFP reappearance at the membrane after egg activation.

It would also be predicted that Ae2-GFP in MII eggs should re-localize if MEK/MAPK is inhibited with U0126. However, U0126 parthenogenetically activates eggs [15], [47]. In that setting, any reappearance of Ae2-GFP in the membrane could not be independently attributed solely to MEK inactivation, in view of our previous demonstration that Ae2 relocalizes to the membrane after egg activation in Sr^2+^-activated eggs. Thus, we did not attempt this experiment.

## Discussion

pH_i_-regulatory mechanisms, including HCO_3_^−^/Cl^−^ exchange, are inactivated during meiotic progression of mouse and hamster oocytes, and then reactivated after egg activation [10], [11], [28], [32], but the mechanism controlling these changes in activity had been unknown. Here, we have found that inactivation and reactivation of HCO_3_^−^/Cl^−^ exchange during meiosis appears to result from changes in Ae2 polypeptide membrane localization controlled by MEK/MAPK activity.

Ae2 is the primary AE family HCO_3_^−^/Cl^−^ exchanger expressed in mouse eggs [28], [29]. Ae2 immunostaining at the membrane was strong in GV oocytes but essentially absent in MII eggs, indicating loss of endogenous Ae2 from the vicinity of the plasma membrane during meiosis. Plasmalemmal localization of Ae2 in GV oocytes but not in MII eggs was further supported by imaging functional GFP-tagged Ae2 in live oocytes, allowing quantitative demonstration of Ae2-GFP loss from the membrane during progression from the GV to MII stages. This loss paralleled a decrease in measured HCO_3_^−^/Cl^−^ exchanger activity due to exogenous Ae2-GFP.

Ae2 loss from the membrane following meiotic maturation was not reflecting a general property of transmembrane proteins in mouse oocytes, since hD1-GFP exogenously expressed in oocytes remained localized to the membrane as the oocyte progressed from GV to MII. Similarly, the activities of several other membrane-localized transporters persist throughout meiotic maturation in mouse oocytes, including the GLYT1 glycine transporter and the TAUT β-amino acid transporter [48], [49], both of which share with Ae2 dependence on Cl^−^.

Ae2 membrane localization was lost between 4 and 8 hours after initiation of meiotic progression (Figure 8A,C), the same period during which HCO_3_^−^/Cl^−^ exchange becomes inactivated [11]. The reappearance of HCO_3_^−^/Cl^−^ exchanger activity after egg activation was similarly associated with re-localization of Ae2-GFP to the membrane. Thus, we propose that a novel feature of meiosis in the mouse oocyte is the selective transient loss of Ae2 protein from the membrane during MI, followed by re-localization of Ae2 to the membrane after egg activation, accompanied by parallel changes in HCO_3_^−^/Cl^−^ exchanger activity.

A key mechanism controlling meiotic cell cycle progression in oocytes is Cytostatic Factor (CSF), a major component of which is the MOS/MEK/MAPK signaling pathway [14], [36], [50], [51]. The major function of CSF is to mediate MII arrest in the egg awaiting fertilization [52]. However, MAPK activity has other functions during the meiotic cell cycle, including maintenance of meiotic spindle structure [14], [47], [50]. Upon egg activation, inactivation of MPF and emission of the second polar body are independent of MAPK, but MAPK inactivation mediates the subsequent appearance of pronuclei [36]. Our results support an additional novel role of MEK/MAPK activity during meiosis in negatively regulating Ae2 membrane localization and HCO_3_^−^/Cl^−^ exchanger activity. Expression of a constitutively active form of MAPK kinase (MEK-EE) that maintained MAPK activity prevented both reactivation of HCO_3_^−^/Cl^−^ exchanger and Ae2-GFP re-localization to the plasma membrane after parthenogenetic egg activation with Sr^2+^. Conversely, expression of a dominant negative MEK (MEK-AA) that significantly inhibited MAPK activity prevented the normal inactivation of HCO_3_^−^/Cl^−^ exchange during meiotic progression. Furthermore, abolition of MAPK activity with the MEK inhibitor, U0126, led to abnormal persistence of Ae2-GFP membrane localization in MII eggs. Thus, the MEK/MAPK pathway is involved in regulating Ae2 localization and activity during meiotic maturation.

Future studies are needed to elucidate the mechanisms by which MAPK activity controls Ae2 localization. Possibilities are endocytosis of the Ae2 protein during meiotic maturation with reinsertion into the plasma membrane after egg activation, or alterations in dynamic recycling at the plasma membrane resulting in a shift of the Ae2 population away from the membrane during maturation. Both are consistent with the observation that de novo protein synthesis is not required for the reappearance of HCO_3_^−^/Cl^−^ exchanger activity after egg activation [28]. Although not previously shown in mammals, this would resemble the reported endocytosis-mediated downregulation of sodium pumps, Ca^2+^-ATPase, and several other ion transporters in by endocytosis during meiotic maturation of *Xenopus* oocytes [53]–[56].

The physiological role of inactivation of HCO_3_^−^/Cl^−^ exchange and removal of Ae2 from the membrane remains unknown. In oocytes of Xenopus [57] and C. elegans [58], vesicular trafficking of proteins to and from the plasma membrane regulates GV arrest. However, MII eggs overexpressing Ae2-GFP exhibited HCO_3_^−^/Cl^−^ exchanger activity as high as that in GV oocytes or fertilized eggs (see Figure 6B) without perturbation of meiotic progression. Thus, changes in HCO_3_^−^/Cl^−^ exchanger activity are not required for normal meiotic progression.

A possible function of HCO_3_^−^/Cl^−^ exchange during meiosis would be to mediate pH_i_ change. In a number of invertebrates including the classic example of sea urchin, increased pH_i_ is required for egg activation [59]. However, pH_i_ does not change significantly in mouse oocytes during meiosis or egg activation [60], [61], appearing to rule out such a role. The other major role for HCO_3_^−^/Cl^−^ exchange, and for Ae2 in particular, is in cell volume regulation. Here, Ae2, functionally coupled to Na^+^/H^+^ exchange (NHE family) mediates the uptake of Na^+^ and Cl^−^ in response to a cell volume decrease [8], [30] .

Fertilized mouse eggs regulate their cell volume by a novel mechanism not found in other cells, in which glycine accumulated to high levels via the GLYT1 transporter provides intracellular osmotic support and controls cell size [62], [63]. We recently found that this volume regulatory mechanism is first activated during meiosis [48], at approximately the same time that HCO_3_^−^/Cl^−^ exchange is inactivated. Furthermore, Na^+^/H^+^ exchange is not detectable in MII eggs of hamster [10] and mouse (our unpublished data), like HCO_3_^−^/Cl^−^ exchange. It is tempting to speculate that HCO_3_^−^/Cl^−^ exchange and Na^+^/H^+^ exchange are both downregulated during meiosis to inactivate Na^+^- and Cl^−^-dependent cell volume regulation while glycine, rather than NaCl, is preferentially accumulated in the egg. However, this hypothesis remains to be investigated.

In summary, our results indicate that HCO_3_^−^/Cl^−^ exchange and Ae2 localization are regulated downstream of MEK/MAPK activity during mouse oocyte meiotic maturation and egg activation. This novel action of MEK/MAPK provides the first example during mammalian meiotic maturation of functional regulation of ion transport by control of membrane localization.
